# Calculated thickness dependent plasmonic properties of gold nanobars in the visible to near-infrared light regime

**DOI:** 10.1371/journal.pone.0177463

**Published:** 2017-05-09

**Authors:** Pijush K. Ghosh, Desalegn T. Debu, David A. French, Joseph B. Herzog

**Affiliations:** 1 Department of Physics, University of Arkansas, Fayetteville, Arkansas, United States of America; 2 Department of Electrical Engineering, University of Arkansas, Fayetteville, Arkansas, United States of America; Institute of Materials Science, GERMANY

## Abstract

Metallic, especially gold, nanostructures exhibit plasmonic behavior in the visible to near-infrared light range. In this study, we investigate optical enhancement and absorption of gold nanobars with different thicknesses for transverse and longitudinal polarizations using finite element method simulations. This study also reports on the discrepancy in the resonance wavelengths and optical enhancement of the sharp-corner and round-corner nanobars of constant length 100 nm and width 60 nm. The result shows that resonance amplitude and wavelength have strong dependences on the thickness of the nanostructure as well as the sharpness of the corners, which is significant since actual fabricated structure often have rounded corners. Primary resonance mode blue-shifts and broadens as the thickess increases due to decoupling of charge dipoles at the surface for both polarizations. The broadening effect is characterized by measuring the full width at half maximum of the spectra. We also present the surface charge distribution showing dipole mode oscillations at resonance frequency and multimode resonance indicating different oscillation directions of the surface charge based on the polarization direction of the field. Results of this work give insight for precisely tuning nanobar structures for sensing and other enhanced optical applications.

## Introduction

When light illuminates metal nanostructures, the free electron gas density oscillates collectively. This collective oscillation is known as a *surface plasmon* or simply a *plasmon*. Strong local-field enhancements, light absorption, and scattering all occur at a resonant incident wavelength, which can depend on the polarization of the light [1–3]. Due to their small volume, gold nanobars exhibit little radiation damping [1]; as a result, they show large local-field enhancement factors and large light scattering efficiency. Because of these characteristics, nanorods prove interesting for optical applications [1]. Nanorods can be used as nanoantennae and have been shown to be useful for many applications such as in enhancing light-emitter interactions [4,5], high-resolution microscopy and spectroscopy [6], optical sensors [7–10], plasmonics in THz range [11], solar cells [12–18], and photocurrent generation [19].

Others have both computational and experimentally investigated plasmonic properties of various metal nanostructures [20] including gold nanowires [21], nanobars [22], nanorods [23]-[24], nanorod arrays [25], nanodiscs [25,26], gold dimers [27], triangular silver prisms [28], nanocuboids [29], nanostar [30] and hybrid or heterostructures [31,32]. These works show that plasmonic properties depend on size, shape, material, and dielectric environment [30,33]. Previous studies have investigated length [34], aspect ratio [35], polarization of incident light [36], and nanostructure fabrication method [37]. Various methods have been used to evaluate the plasmonic properties, such as discrete dipole approximation [38], quasistatic approximation [39], etc. While much work investigates the plasmonic properties in nanorods that have a circular cross-section, here we investigate the plasmonic properties of Au nanobars that have a rectangular cross-section and carefully examine the effects of their thicknesses using a finite element method. Importantly, we explore the differences between sharp-corner nanobars and more realistic round-corner nanobars in terms of the resonance wavelength and optical enhancement as the thickness is varied.

## Methods

**Fig 1:**
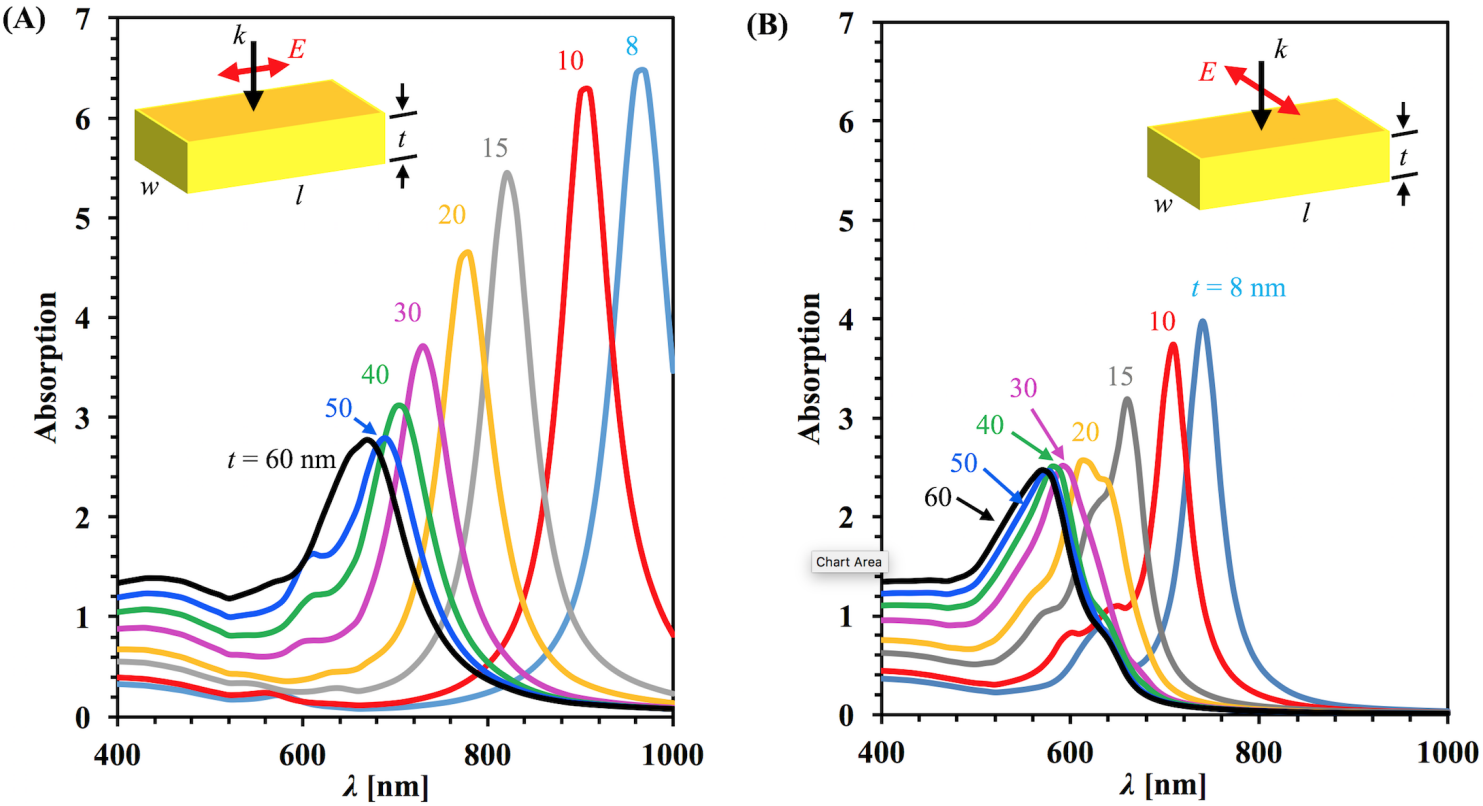
Calculated absorption spectrum. For the sharp-corner rectangular gold nanobar with different thickness (A) For longitudinal polarization and (B) for transverse polarization. In both cases light is normally incident. Insets show to top view of the nanobar with polarization direction.

Computational analyses were performed on gold nanobars of constant length and width with different thicknesses using COMSOL simulations. The simulations are performed in three-dimensional space, where the length of the nanobar is 100 nm, the width is 60 nm, and the thickness varies from 8 nm to 60 nm. The geometries are chosen to represent nanobar structures that can be fabricated with electron beam lithography on a silicon substrate with a silicon dioxide layer. The substrate effect was approximated by an effective medium *n*_*eff*_ = 1.25 around the nanobar [40–43]. For gold, the optical properties are obtained from Johnson and Christy [44]. A normally incident light was directed onto the surface of the nanobar with the electric field polarized along either the longitudinal or transverse direction as shown in the insets of Fig 1. For the simulation, two types of rectangular nanobars, sharp-corner and round-corner, were investigated. The round-corner bars have a 15 nm fillet on the vertical edges. Absorption of the nanobars was calculated using the heat loss in the volume of the nanobars. The optical enhancement, defined as the ratio of the local electric field ***E*** to the incident electric field ***E***_0_ squared (*E*^2^*/E*_0_^2^), was studied since light intensity is proportional to the electric field squared. Around the sample, an integration space of radius 125 nm has been defined for the near-field region where most of the enhancement occurs. This integration space was used to calculate the optical enhancement of the nanobar.

## Results and discussion

The absorption spectra of the nanobars was calculated for each thickness variation and is plotted in Fig 1 for both longitudinal (A) and transverse (B) incident polarizations. Each line on the plot indicates the spectrum of a single nanobar with a particular thickness. The plot shows that the absorption peak shifts toward blue as the thickness increases for both polarizations. For a given thickness, the large peak in the spectrum corresponds to the dipolar resonant plasmonic mode, and the smaller peaks at the lower wavelenghts are due to higher order modes. This is illustrated and discussed more later with our charge distribution calculations. These results are consistent with previous results on nanobars [29].

For the transverse polarization, the observed trend is same but the amplitude is reduced compared to the longitudinal polarization. The resonance peak value comparing the two polarizations for the same geometrical parameters gives different position. For longitudinal polarization, the resonance wavelength value is larger than for the transverse polarization because plasmonic response depends on the geometric length along which the electric field is polarized [45]. The full-width at half maximum of the spectrum also increases with increasing thickness.

**Fig 2:**
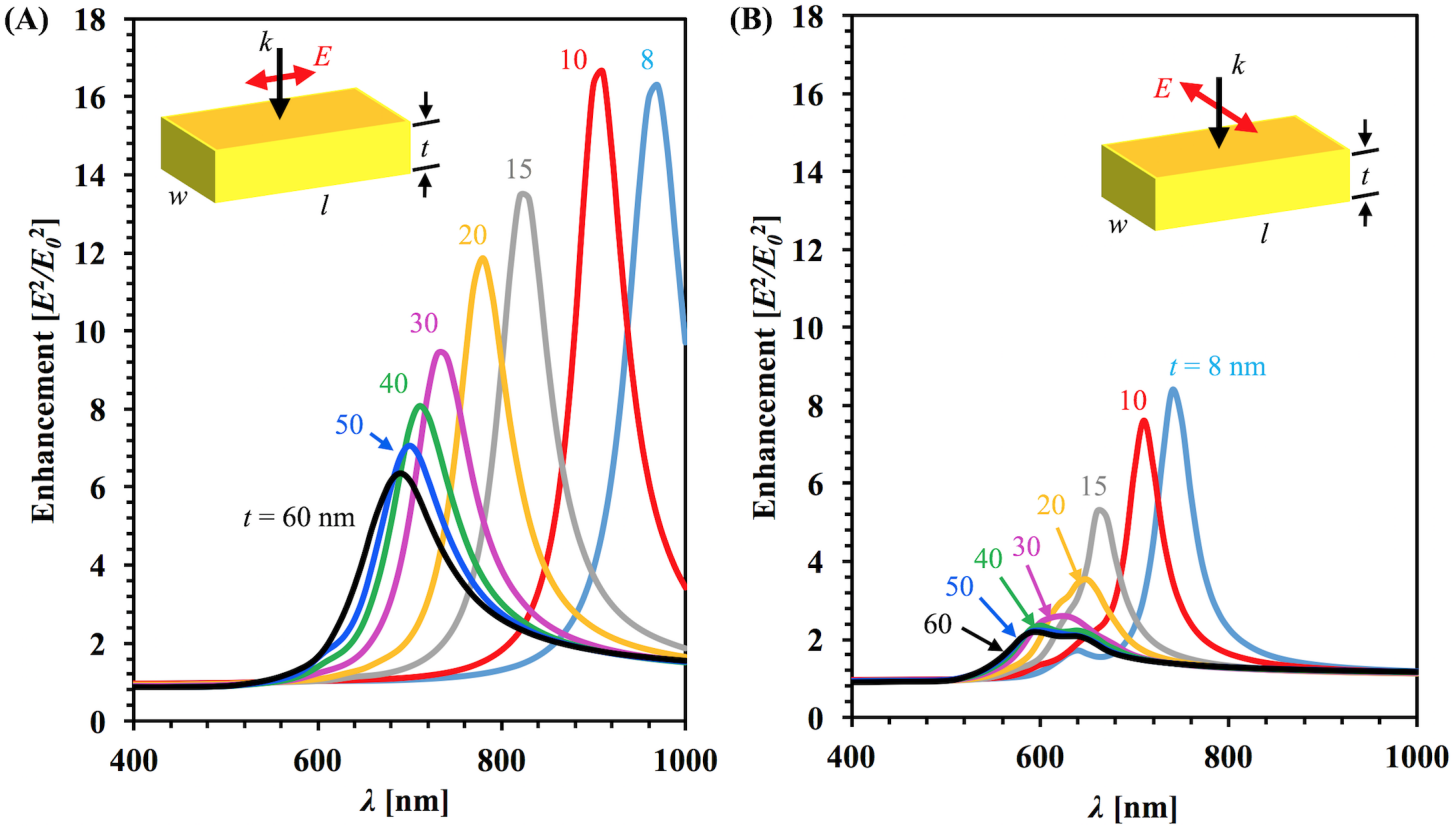
Calculated average enhancement spectrum. Spectrum was calculated in the integrated volume of the sharp-corner rectangular gold nanobar for normal light incidence in the effective medium (*n*_*eff*_ = 1.25) with different thickness for (A) longitudinal polarization (B) transverse polarization.

Fig 2 shows the optical enhancement spectra for nanobars of constant length and width with different thicknesses for both longitudinal (A) and transverse (B) polarizations. Again the enhancement peak shifts towards blue with increasing thicknesses. Many studies looking at the length of the nanobars have found that increasing the length of the nanobars shifts the resonance peaks towards the red [4,24]. This difference arises due to the direction of the k-vector. When the k-vector is perpendicular to the dimension of interest, such as length, then the previous results hold true. For a k-vector parallel to the dimension of interest, the peaks blue-shift as thickness increases. For the same gold thickness, the peak resonance wavelength and normalized amplitude exhibit significant differences due to the different polarizations for both absorption and enhancement. For polarization along the long axis, electrons oscillate in a larger space, taking a longer time to oscillate, causing a lower frequency, and as a result the peak resonance wavelength is longer than polarization along the short axis of the nanobars [46].

**Fig 3:**
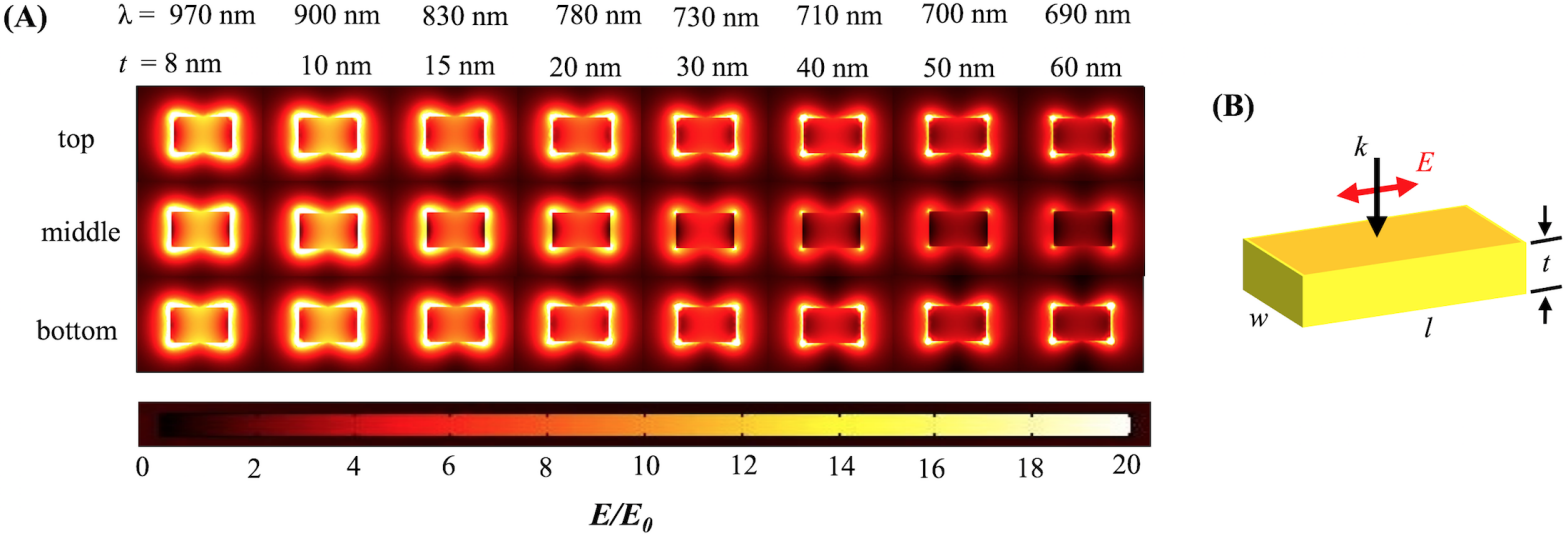
Electromagnetic field distributions for sharp-corner nanobar. (A) Field distribution for top, middle, and bottom surfaces at resonance wavelengths for sharp-corner Au nanobars of length 100 nm and width 60 nm for different thickness when polarization is aligned along the long axis and normal incidence. (B) Schematic of sharp-corner nanobar.

**Fig 4:**
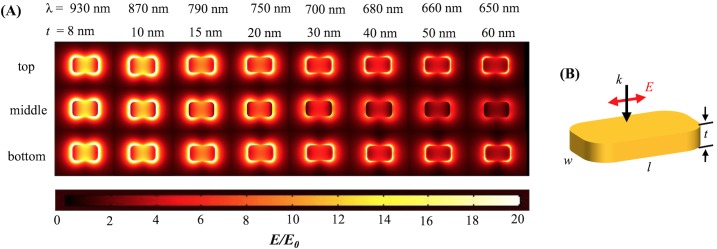
Electromagnetic field distributions for round-corner nanobar. (A) Field distribution for top, middle and bottom surfaces at resonance wavelengths for round-corner Au nanobars of length 100 nm and width 60 nm for different thickness when polarization along the long axis and normal incidence. (B) Schematic of round-corner nanobar.

The electric field distribution (***E/E***_***0***_) is shown at the resonant wavelength for each thickness for the sharp-corner and round-corner rectangular gold nanobars in Figs 3(A) and 4(A) respectively. The maximum amplitude of the electric field distribution at the resonant wavelength decreases as the thickness increases. This is due to the top and bottom plasmons decoupling for large thicknesses.

**Fig 5:**
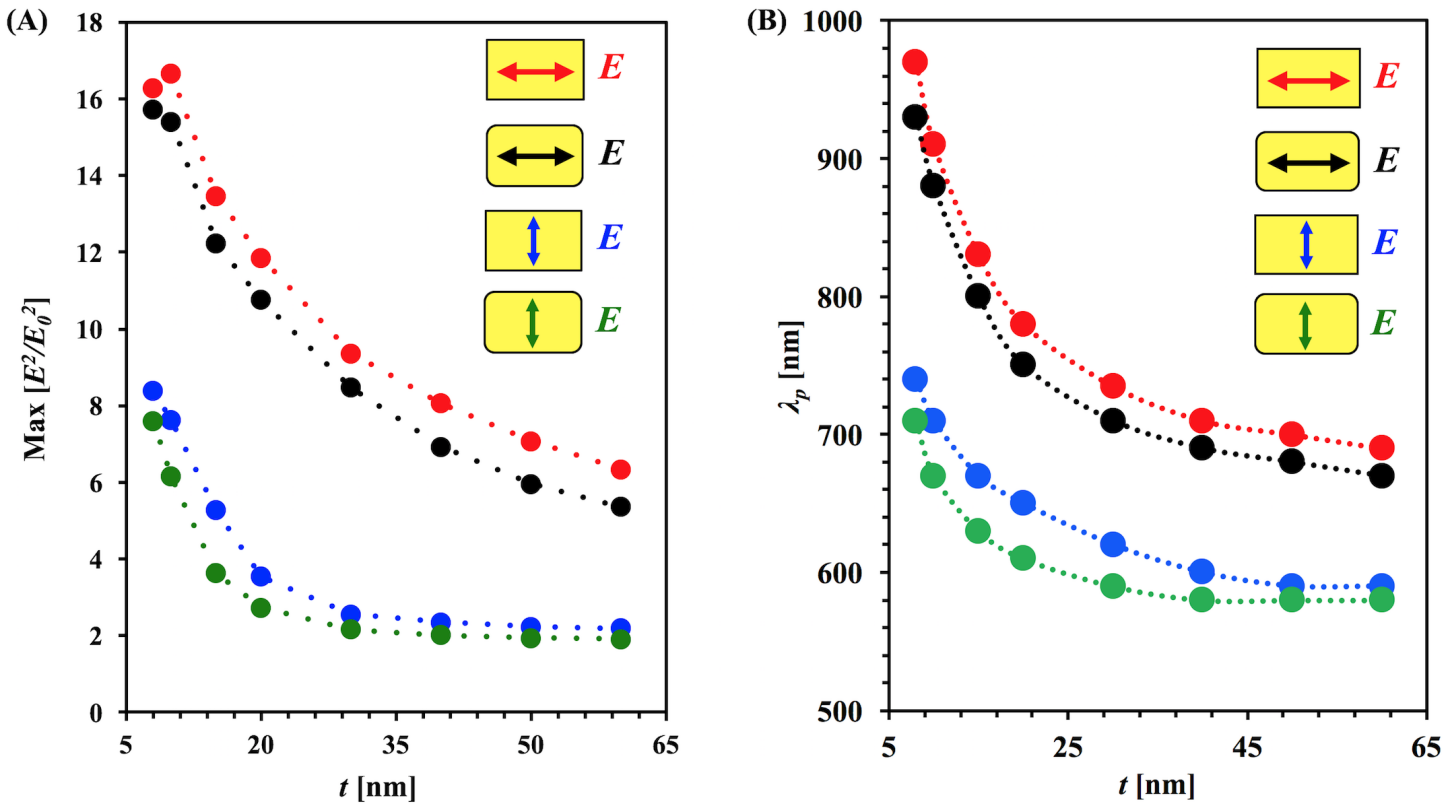
Dependance of maximum enhancement and peak resonance wavelength on thickness. (A) Normalized maximum enhancement [arb. unit] at resonant incident wavelength as a function of thickness for sharp-corner and round-corner nanobars for longitudinal and transverse polarization (B) Peak resonance wavelength as a function of thickness for both longitudinal and transverse polarization.

Fig 5(A) shows the maximum electric field enhancement of sharp-corner and round-corner structures under longitudinal and transverse polarizations of incident light as a function of thickness. Maximum enhancement decreases with increasing thickness. The sharp-corner structures have a maximum enhancement that is between 3–18% greater than round-corner structures for longitudinal polarization with and average of a 12% increase, and the enhancement increase ranges from 10–46% with an average of 21% for transverse polarization. In practice, nanobars fabricated with lithography will have rounded corners due to the finite radius of the electron beam. For the longitudinal polarization, the maximum enhancement decreases quickly and tends toward a stable value at higher thickness. For transverse polarization for both shapes, the maximum enhancement decreases quickly up to 20 nm thickness; after that, the change becomes significantly less and eventually the maximum enhancement reaches a constant value. For large thicknesses, the plasmon modes are dominated by the dipole mode of the surface charges; those are well distributed on the surface of the nanoparticle. In this case, the surface charges are not strongly confined, creating only a weak enhancement. In case of small thicknesses, the resonance includes higher modes along with the dipole mode. The dipole mode’s polarization source charges are well confined around the corners and edges of the nanobar, creating localized strong hot spots that generate strongly enhanced fields. The sharp corner nanobar accumulates more charges in the corners than round corner nanobars which makes a greater difference in the electric field enhancement. Additionally, increases in free electron density cause a blue shift of the surface plasmon resonance due to the enhanced restoring force [47].

Fig 5(B) shows the peak resonance wavelength for the enhancement spectrum as a function of thickness. As the thickness increases, the peak resonance wavelength shifts toward blue for both polarizations for both sharp-corner and round-corner nanobars. Additionally, the resonant wavelength for the round-corner structures is blue-shifted by 10 to 40 nm relative to the sharp corners. The nanobars with round corners have a reduced effective dimension along the side with the round corners which contributes to the blue-shift. Another contribution is due to the charge distribution; in round-corner nanobars, charges spread more than sharp-corner nanobars, which leads to a blue shift [48]. This is an important factor to consider when designing and tuning nanobars for plasmonic applications.

**Fig 6:**
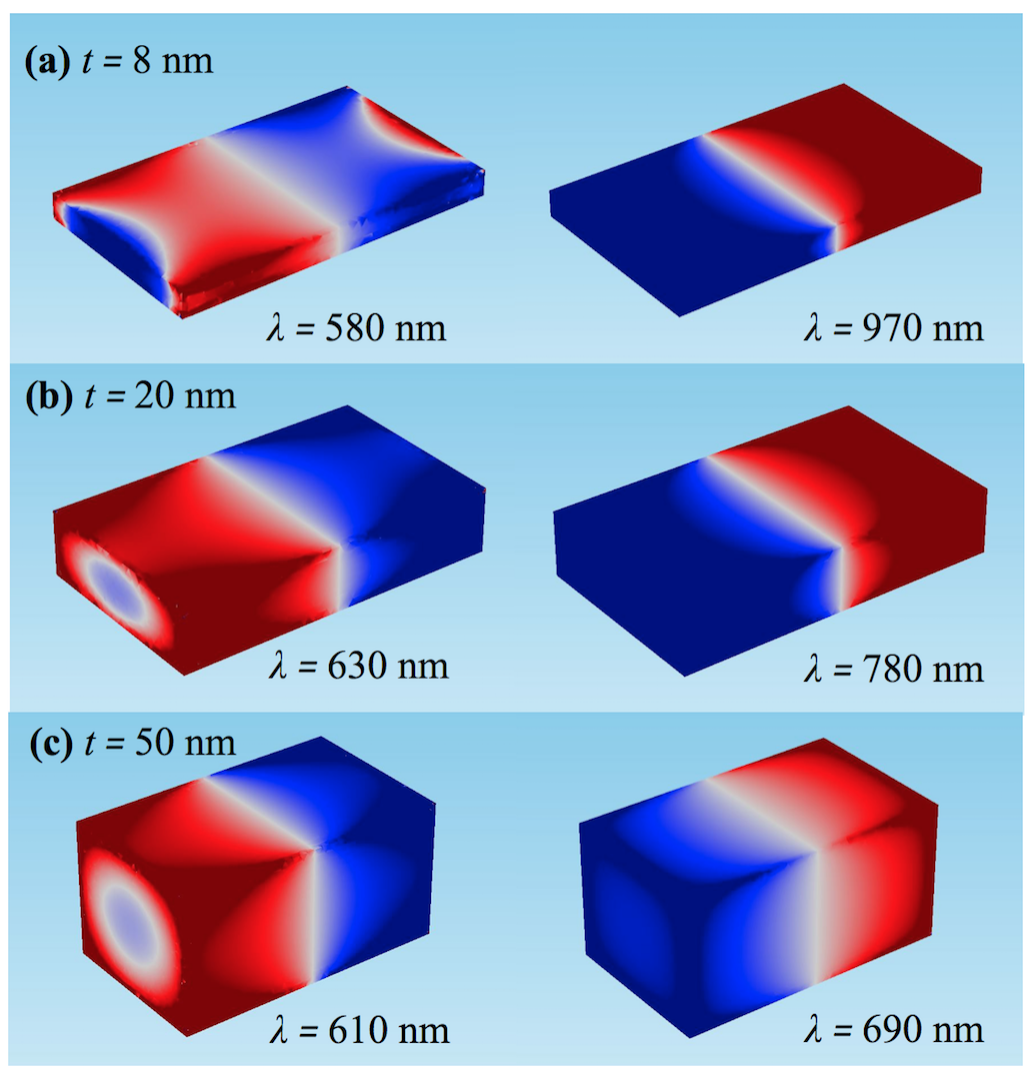
Surface charge distributions. At peak resonance wavelength when the thickness is (a) 8 nm (b) 20 nm and (c) 50 nm.

Fig 6 demonstrates the surface charge distributions for three different thicknesses for longitudinal polarization. Using Gauss’s law (Eq 1) surface charge was calculated through the metal surface, *S* [49].

$$Q=n_{eff}^{2}\cdot \varepsilon_{0}∯\left(n\cdot E\right)dS=n_{eff}^{2}\cdot \varepsilon_{0}∯\left(n_{x}\cdot E_{x}+n_{y}\cdot E_{y}+n_{z}\cdot E_{z}\right)dS$$ (1)

Fig 6(A) shows the surface charge distribution for a nanobar with a thickness of 8 nm. At the resonance wavelength 580 nm the charge distribution forms a quadrupole while at 970 nm there is a dipole moment. Similar behavior was found for 20 nm and 50 nm thickness. These wavelengths reflect the two different peaks shown earlier in Fig 1. At the primary resonance wavelength, there is a dipolar distribution while at the shorter, lower intensity peak there is a quadrupolar distribution.

For small thickness, the dipole on the top surface and the dipole on the bottom surface are strongly coupled to each other and have a larger effective total charge. As the thickness increases, these dipoles begin to decouple and act more as separate dipoles. This causes a decrease in the total effective oscillating charge, thereby increasing the oscillation frequency, resulting in a blue shift. In addition, as the charges decouple, the higher order modes become more prominent. This change in relative intensities between modes, along with the primary resonant peak blue-shifting due to the higher orders, results in a broader spectral peak.

**Fig 7:**
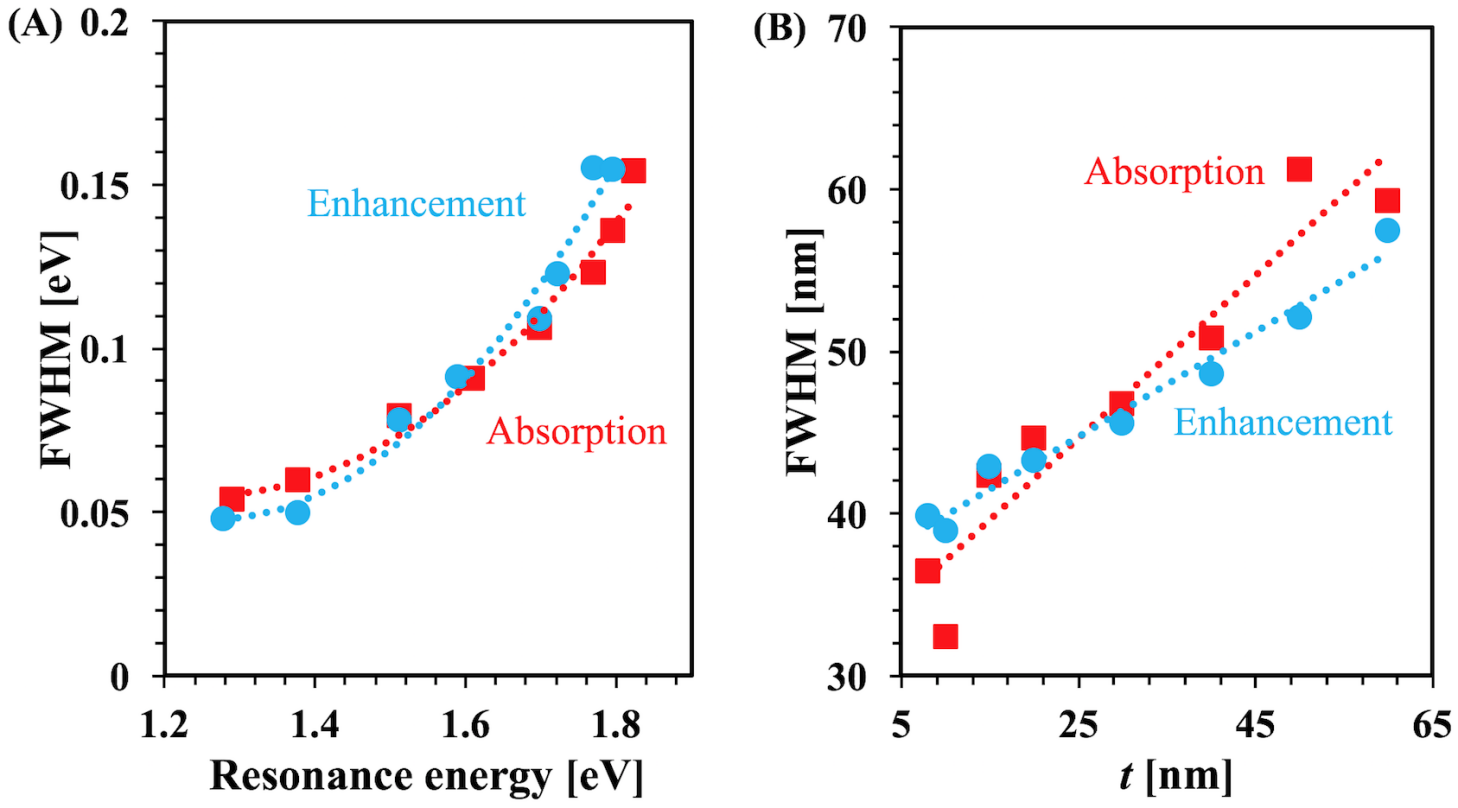
Resonance energy and thickness dependend FWHM. (A) Full width half maximum as a function of resonance energy (B) Full width half maximum as a function of thickness, where light incident normally.

Fig 7(A) plots the full-width at half maximum (FWHM) as a function of resonance energy for each geometry. For absorption and enhancement, the figure shows nonlinear behavior with resonance energy. Fig 7(B) plots the FWHM of the broadening of the enhancement and absorption spectra as a function of thickness. For both cases, the plots show that FWHM generally increases with thickness; this is in part due to the superposition of the dipolar mode with higher order modes at lower wavelegnths.

The enhancement and absorption spectra broaden with the thickness of the structure. Fig 7(A) shows an interesting result; when the thickness is changed, FWHM increases with the nanobar thickness. It shows a similar trend to that in Y-H Qiu et al. [23] where plasmon width was plotted as a function of longitudinal surface plasmon resonance (L-SPR) energy as the gold nanobar length was changed. This broadening is because of interband excitation induced damping [23]. In plasmon relaxation dynamics, a three-level system was introduced by Y-H Qiu et al. According to this system, a fixed frequency is used to excite the electron from ground state to the second excited state. A two-step process is used by electrons to go to the ground state; first, the electrons transition from second excited state to the first excited state then from first excited state to the ground state. From the second excited state to the first excited state, the electron goes through a process called electron-phonon relaxation, and from the first excited state to the ground state, the electron transitions with decay rate due to the phonon-phonon relaxation process.

## Conclusion

In this study, plasmonic properties of gold nanobars with various thicknesses were computationally analyzed. Sharp-corner and round-corner rectangular nanobars were studied; comparisons of the peaks the spectra reveal that the round-corner nanobars shows an average of 30 nm shift to lower wavelength for the same size for both incident polariztations. Resonance peak wavelengths shifted toward blue for both absorption and enhancement spectra with increasing thicknesses due to decoupling of the charge dipoles on the top and bottom surface. As the thickness increases, the FWHM of the spectrum also increases due to the impact of higher order modes which have been visualized in our surface charge density distrubution calculations. The results reported here are significant for plasmonic structures fabricated with elelectron beam lithography since we investigate key fabrication parameters including thickness of the nanobars and round corners, a property of nanostructures fabricated with method.

## Supporting information

S1 File Data file for sharp corner structures. (XLSX)

S2 File Data file for rounded corner structures. (XLSX)
